# Long-Term Modeling and Monitoring of Neuromusculoskeletal System Performance Using Tattoo-Like EMG Sensors

**Published:** 2019

**Authors:** Kaiwen Yang, Luke Nicolini, Irene Kuang, Nanshu Lu, Dragan Djurdjanovic

**Affiliations:** 1 The University of Texas at Austin, Austin, TX,78712, USA; 2 Massachusetts Institute of Technology, Cambridge, MA, 02139, USA

## Abstract

This paper introduces stretchable, long-term wearable, tattoo-like dry surface electrodes for highly repeatable electromyography (EMG). The tattoo-like sensors are hair thin, skin compliant and can be laminated on human skin just like a temporary transfer tattoo, which enables multi-day noninvasive but intimate contact with the skin even under severe skin deformation. The new electrodes were used to facilitate a system-based approach to tracking of long-term fatiguing and recovery processes in a human neuromusculoskeletal (NMS) system, which was based on establishing an autoregressive moving average model with exogenous inputs (ARMAX model) relating signatures extracted from the surface electromyogram (sEMG) signals collected using the tattoo-like sensors, and the corresponding hand grip force (HGF) serving as the model output. Performance degradation of the relevant NMS system was evaluated by tracking the evolution of the errors of the ARMAX model established using the data corresponding to the rested (fresh) state of any given subject. Results from several exercise sessions clearly showed repeated patterns of fatiguing and resting, with a notable point that these patterns could now be quantified via dynamic models relating the relevant muscle signatures and NMS outputs.

## 1. Introduction

Even if the study of electromyogram (EMG) can be traced back to 1666 and has been a major research topic ever since, very few characteristics of surface electromyogram (sEMG) signals are used in fatigue detection and performance analysis. The reason is that sEMG is notoriously noisy and susceptible to noise from surface tissues (Kuiken *et al*. 2003). Furthermore, dynamic body motion like walking and running can cause inconsistency of the connection and detachment of electrodes. On the other hand, researchers have shown connections between changes in certain characteristics of EMG signals and muscle fatigue in both the time and frequency domains. E.g., Gerdle *et al*. (2000) use changes in the amplitudes of EMG signals to detect fatigue, while Dimitrov *et al*. (2006) use spectral (frequency domain) indices to track fatigue before and after maximum knee torque. Signatures extracted from joint time-frequency distributions EMG signals have also been used as indicators of fatigue, including the instantaneous frequencies (Allison & Fujiwara, 2002) and intensities (Disselhorst-Klug *et al*. 2009).

In this paper, a long-term wearable, tattoo-like stretchable sEMG sensor is developed, manufactured and utilized to collect sEMG signals for model-based performance monitoring of a human neuromusculoskeletal (NMS) system. The stretchable sensors are dry, noninvasive, hair thin, skin soft, and capable of long-term biopotential monitoring (Yang, *et al*.,2015; Kabiri *et al*., 2017; Wang *et al*., 2018; Yamagami *et al*.,2018.). The subjects are able to wear this tattoo-like sensor for days freely, without any irritation or other discomfort. In such a way, these tattoo-like sensors enable a long-term system-based investigation of muscle fatigue and recovery over time by solving the problem of inconsistent electrode positioning between trials, as well as the sensor detachment.

The vast majority of research on modeling and monitoring of human NMS system performance is symptom-based, meaning that the NMS condition is characterized via analysis of relevant signals, such as joint angles and/or angular velocities, electrical activities in muscles (EMG), limb and/or reaction forces, etc. A symptom-based monitoring paradigm, such as what we see in the Phase Space Wrapping method (Dingwell *et al*. 2007), Goal Equivalent Manifold (GEM) approach (Gates & Dingwell, 2008), or limb force-based NMS analysis (Vøllestad, 1997) is valid only when the system input is stationary (Musselman *et al*. 2017), and this is not true during regular operation of most complex systems, including (especially) biological ones.

Functionally speaking, various portions of the NMS system can be seen as dynamic systems for which inputs are the neurally-induced electrical activities in the relevant muscles, visible in the EMG signals, while joint and limb movements and forces can be seen as outputs (Keynes *et al*. 2001). Therefore, the machine-monitoring technique based on establishing and tracing dynamic models of inputs and outputs, rather than purely signal-based monitoring techniques, can be employed to monitor NMS system performance during its regular activities. Thus, in addition to the use of the novel tattoo-like long-term sEMG sensors, the research presented in this paper will utilize a system-based monitoring paradigm to track human body fatigue over longer periods of time (days).

The remainder of the article is structured as follows. In Section 2, the “cut and paste” based manufacturing technique and design of tattoo-like sensors specifically for the experiment are described. Section 3 describes the methods for EMG signal processing, modeling of NMS system dynamics, and model-based tracking of changes in the NMS system performance. Section 4 presents results of applying the tattoo electrode based EMG data collection system and the model-based NMS performance method to fatigue characterization and tracking in forearm portion of the NMS system of multiple subjects, over multiple exercise sessions and days. Finally. Section 5 summarizes findings of the research presented in this paper and outlines avenues for possible future work.

## 2. Sensor Manufacturing, design, and performance

### 2.1 Cut-and-Paste Method

Creation of dry and stretchable tattoo-like electrodes requires precise shaping of gold nanomembranes in order to create patterns that are conductive, stretchable, and conformable enough to operate on the human skin. Traditional nanomanufacturing, the industry standard for the creation of ultrathin devices, is labor and equipment intensive process, usually involving many different steps and the use of many different chemicals, all of which add up to a significant cost in time and money. Thus we have invented a unique manufacturing method dubbed “Cut-and-Paste” method, which uses entirely dry and freeform (no mask or template) processes with countertop operation in order to manufacture tattoo-like electronics in only a few minutes (Yang, *et al*.,2015; Kabiri *et al*., 2017; Wang *et al*., 2018; Yamagami *et al*.,2018.). The method is applicable to all types of thin metals, polymer sheets, and even atomically thin two dimensional materials, and creates devices that are shown to accurately measure a wide array of signals, including biopotentials, skin hydration, and surface temperature. In this study, we used such “Cut-and-Paste” method to manufacture tattoo-like sEMG sensors.

### 2.2 Sensor design and deformation test

**Figure 1:**
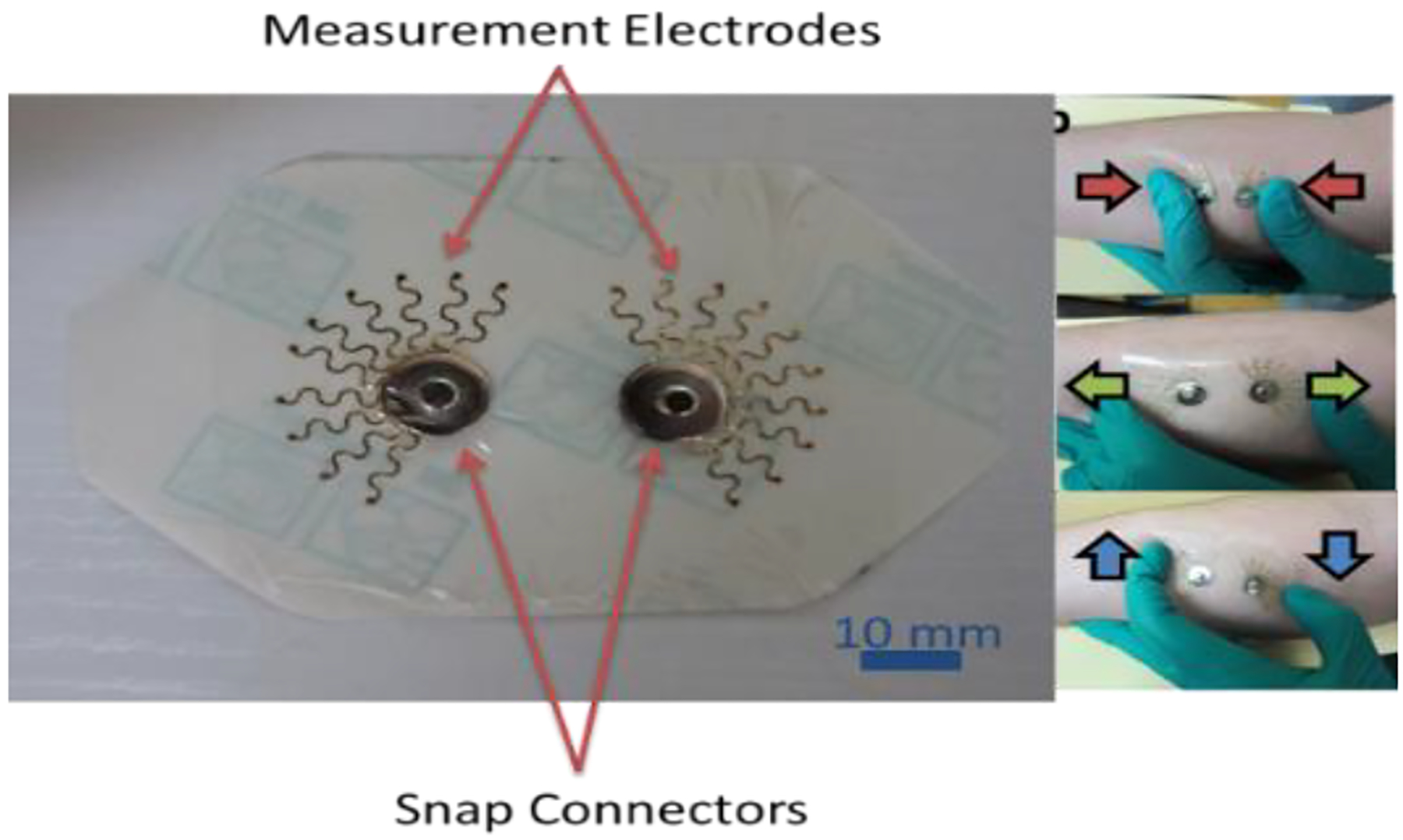
Sun-type sensor design and deformation test on skin.

Due to the nature of muscles to move during contraction and extension, a stretchable sensor was designed to capture EMG signals based on the Cut-and-Paste technology described in previous subsection. This new design is shown in Figure 1, both stand-alone and on the target arm muscles under deformation.

The new design features two electrodes patterned in a “Sun” pattern with meandering “radiance”, to increase electrode surface area, while maintaining stretchability and conformability. The electrodes also each feature a snap-connector, which allows for easy connection of the electrodes to a measurement device during data capture, either by snap-on wire connectors or alligator clips. Tegaderm was chosen as the substrate for the device for its skin-like qualities, good adhesion strength, and stretchability greater than needed for muscle movement during testing (Yang *et al*,2015). To ensure the capabilities of the tattoo-like device under deformation, several deformations were performed on a live subject, including stretching, compression, shearing, and poking. The resistance of the Au serpentines were measured before and after the deformations, and show negligible resistance change as a result of deformation, indicating that the deformations did not affect the electrical capabilities of the device. The stretchability allows both more conformal contact for truer EMG measurements, and also does not constrain the muscle during movement in order to capture the truest possible free muscle dynamics for study.

## 3. System-based Monitoring, fatigue tracking, and performance evaluation

### 3.1 EMG signal processing and feature extraction

Raw surface EMG signals are highly noisy and nonstationary, which presents a great challenge in the signal analysis. On the other hand, various research shows the connection between muscle fatigue and changes in the amplitude and frequency content of EMG signals (Gerdle *et al*., 2000; Dimitrov *et al*., 2006; Allison *et al*., 2006) Cohen’s class of time-frequency distributions (TFD) offers a powerful tool for description and analysis of highly noisy and non-stationary signals, such as EMG signal (Cohen, 1995). A TFD reveals the distribution of signal energy concurrently in both the time and frequency domains by quantitatively describing what portion of signal energy resides at any given time and at any given frequency. More formally, given a signal *s*(*t*), a Cohen’s class TFD, denoted as *C*(*t*, *ω*), is computed as:

$$C(t,\omega )=\frac{1}{4\pi^{2}}.$$

(1) $$\overset{+\infty }{\underset{-\infty }{∭}}s^{*}\left(u-\frac{\tau }{2}\right)s\left(u+\frac{\tau }{2}\right)\phi (\theta ,\tau )e^{-j(\theta (t-u)+\tau \omega )}d\tau dud\theta$$

where:

- $A(\theta ,t)=\int_{-\infty }^{\infty }s^{*}\left(u-\frac{\tau }{2}\right)s\left(u+\frac{\tau }{2}\right)e^{-j\theta u}du$ is the ambiguity function of signal *s*(*t*)
- *ϕ*(*θ*, *τ*) is the kernel which determines the distribution and some properties.

In this paper, we use the binomial kernel, which is a powerful signal-independent kernel in the family of so-called Reduced Interference kernels, with a set of desirable mathematical properties that enable a high-resolution representation of signal over time-frequency domains, and a much faster calculation of TFDs, compared to the signal dependent kernels. (Jeong & Williams,1992). Once the binomial TFD is calculated, for each moment *t* in time, corresponding instantaneous intensity, mean frequency, second order moment and entropy can be extracted from the time-frequency distribution.

Instantaneous intensity of EMG signals is known to be directly related to voluntary muscle force (Potvin,1997), while the instantaneous mean (expected) frequency has been shown to be a common indicator for muscle fatigue (Bilodeau et al. 2003). Because of the time-domain and frequency-domain marginal properties of binomial kernels (Jeong and Williams, 1992), binomial TFD *C*(*t*, *ω*) of an EMG signal can be used to calculate instantaneous signal intensity < *f*^0^|*t* > at any time sample *t* as

(2) $$<f^{0}|t>=\int C(t,\omega )\text{d}\omega$$

while conditional expectation properties of binomial TFDs (Jeong and Williams, 1992) enable calculation of instantaneous EMG frequencies < *f*^1^|*t* > at time sample *t* as

(3) $$<f^{1}|t>=\int \frac{C(t,\omega )}{<f^{0}|t>}\omega d\omega$$

Even though literature does not recognize relation between higher order instantaneous frequency moments of EMG signals, in order to better describe instantaneous frequency characteristics of EMG signals, input vector for the NMS dynamic model also included second order instantaneous moments of EMG signals < *f*^2^|*t* >, calculated as (Cohen, 1995)

(4) $$<f^{2}|t>=\int \frac{C(t,\omega )}{<f^{0}|t>}\omega^{2}d\omega$$

as well as the instantaneous EMG entropy < *S*|*t* >, calculated as (Cohen, 1995)

(5) $$<S|t>=\int \frac{\text{C}(\text{t},\omega )}{f^{0}|t}\text{ ln }\frac{\text{C}(\text{t},\omega )}{<f^{0}|t>}\text{d}\omega$$

### 3.2 ARMAX modeling and model order determination

EMG featured signal $\vec{X}(t)=[<f^{0}\left|t>,<f^{1}\right|t>,<{f^{2}|t>,<S|t>]}^{T}$ as system input and hand grip force signal *F*(*t*) as system output are utilized to train fresh unfatigued NMS system structured by an Autoregressive-Moving Average with Exogenous input (ARMAX) which defines an evolution rule formulated in the following equation:

(6) $$F(t)=\sum_{n=1}^{N}\phi_{n}F(t-n)+\sum_{n=1}^{N-1}\theta_{n}a(t-n)+\Gamma \vec{X}(t-1)$$

where:

- *ϕ*_*n*_ are the autoregressive coefficients;
- *θ*_*n*_ are the moving average coefficients;
- Γ denotes the matrix of coefficients relating EMG signatures with the hand grip force^1^;
- *a*(*t*) – modeling residuals, behaving as a Gaussian white noise with variance $\sigma_{a}^{2}$.

To preserve model accuracy as well as simplicity of the NMS system, model order *N* for each subject/trial was instantaneously produce forces (to preserve causality of the resulting dynamic system). determined using the Akaike Information Criterion (AIC) (Akaike, 1974).

### 3.3 Model-based fatigue tracking

Relevant NMS Model of any given subject is trained using the data from the initial 10–20 seconds of his or her exercise period, during which the NMS performance is believed to be the least degraded (the corresponding subject experiences minimum fatigue). The model learnt in this least degraded state will be referred to as the “fresh model”, and the data used to train it will be referred to as “fresh data”. Let us denote as *P* the distribution of 1-step ahead prediction errors the fresh model produces on the fresh data. As the exercise progresses and new data arrives, distribution of the most recent 1-step ahead prediction errors (most recent modeling errors) produced by the fresh model can be generated. Let us denote this distribution by *Q*_*T*_, where *T* denotes the time interval over which the NMS system performance is evaluated (time interval over which the distribution *Q*_*T*_ of modeling errors produced by the fresh model is evaluated). If the NMS system dynamics in the time interval *T* are the same or similar to those observed on the fresh data, the distributions *P* and *Q*_*T*_ should be similar to each other. However, if the NMS dynamics in interval T are changed compared to those observed on the fresh data, due to e.g. fatigue or injury, the distribution *Q*_*T*_ will be different from the fresh distribution *P* and this difference can be quantified and used to track the degradation of NMS performance.

Following, (Musselman et al., 2017), let us quantify the difference between distributions *P* and *Q*_*T*_ via a Fatigue Index defined using the Kullback-Leibler divergence:

(7) $$\text{Fatigue Index }(FI):=D_{KL}\left(P\|Q_{T}\right)=\sum_{i}P(i)\cdot ln\frac{P(i)}{Q_{T}(i)}$$

The non-negative measure FI depicts the similarity between distributions *P* and *Q*_*T*_, with FI close to zero indicating that the current NMS model is similar to the fresh model, while a larger FI implies a more significant difference between the two distributions.

## 4. Results and Discussion

### 4.1 Experiment Setup

The study presented in this paper involved 3 types of exercises (trials), involving periodic squeezing and releasing of a force sensor and resting in between exercise sessions. We will refer to these trials as the Same-day Repetitive Trial (SRT), Multi-day Repetitive Trial (MRT) and Repetitive Fatigue and Recovery Trial (RFRT). During SRT, subject is asked to grip the force sensor with MVC (Maximum Voluntary Contraction) for 2 minutes, then rest for 20–30 minutes until he/she experienced no soreness in the affected muscles. At that point, the subject performed another grip trial for the same duration, followed by a rest for another 20–30 minutes. This fatigue-and-rest process is repeated 8 times. During the MRT, the subject is asked to grip the force sensor for 2 minutes with MVC, after which he/she rested for 20–30 minutes, until subject experienced no soreness, followed by another 2 minutes of gripping the force sensor. The same experiment is performed for 2 more consecutive days. During the RFRT, subject is asked to grip the force sensor with MVC for 2 minutes, after which over the next 30 minutes, the subject is asked to grip the force sensor with MVC for 10 seconds every 2 minutes (this enabled us to track his/her recovery process). This process is repeated twice after which the subject performed a final 2 minutes gripping trial to end the RFRT.

**Figure 2:**
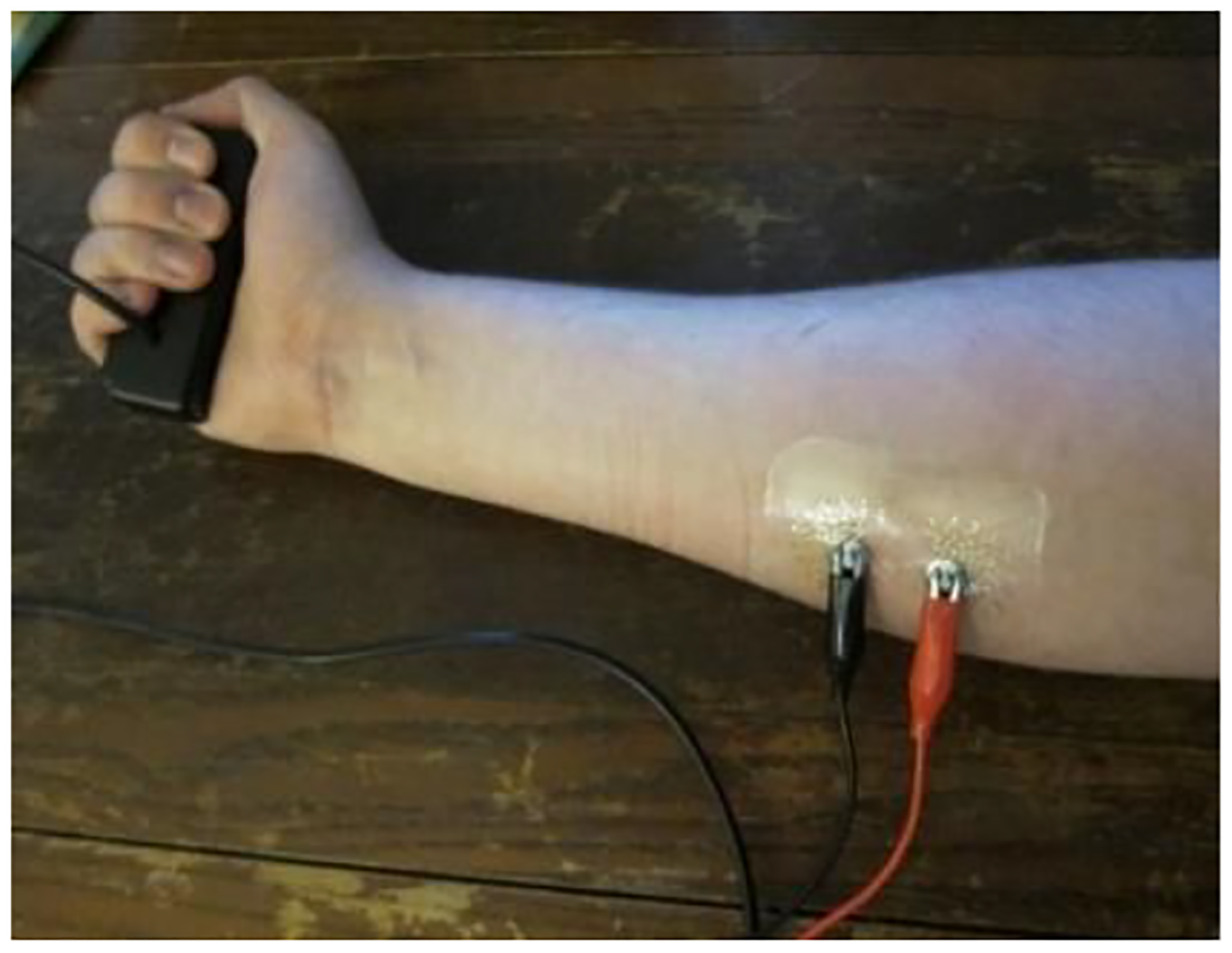
Experimental Setup. Subject grips a commercially available hand dynamometer, while the tattoo-like sensor records their forearm EMG signals.

**Figure 3:**
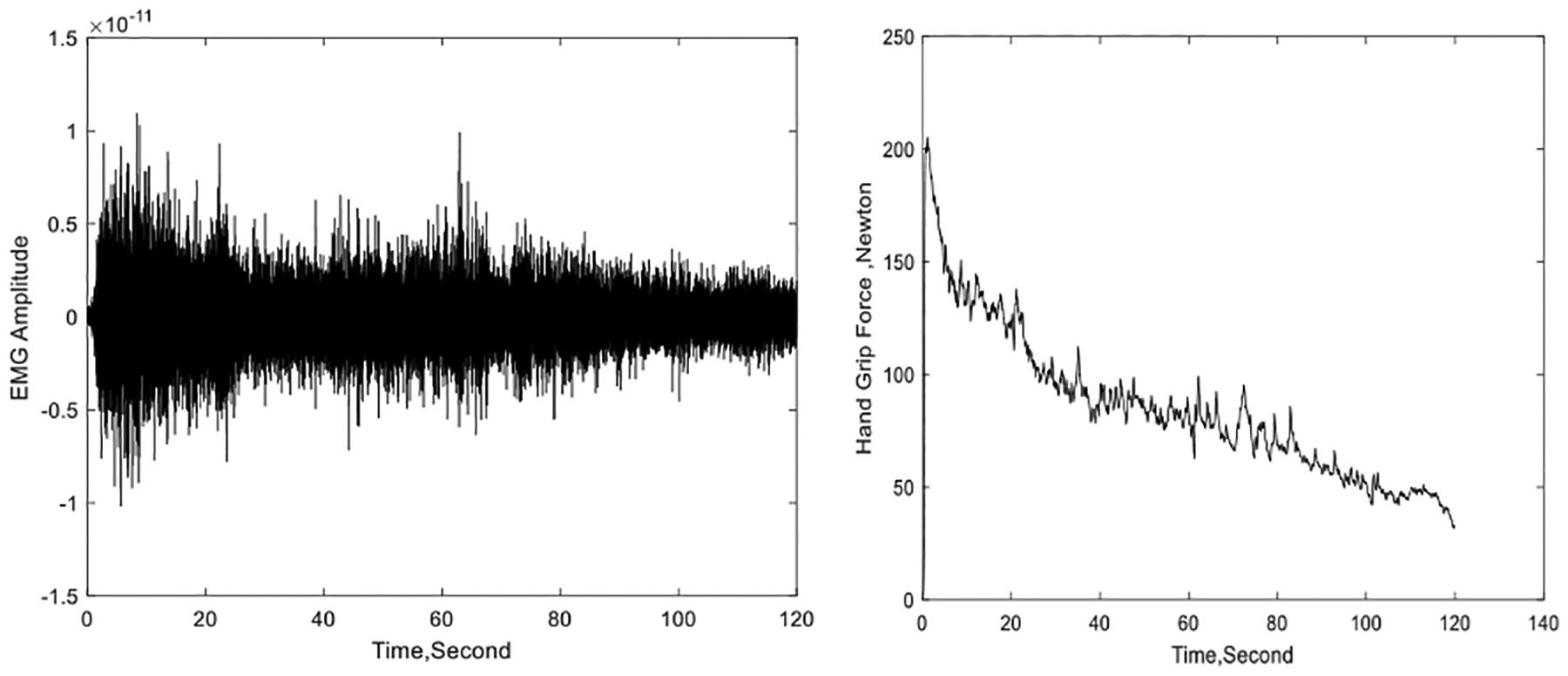
An example of concurrently collected and synchronized EMG (left) and hand grip force (right) signals.

All experiments involved the dominant hand of the relevant subject and the corresponding forearm flexor muscles. The forearm EMG signals were collected using the Sun type tattoo-like sensor described in Section 2, while the HGF data is collected by a bar-shaped hand dynamometer. A signal generator was used to produce pulse signals with a frequency of 100mHz, which were used as timing signals for synchronization of the EMG and hand force datasets. Experimental set up is shown in Figure 2, while an example of concurrently collected and synchronized EMG and hand force signals is shown in Figure 3.

### 4.2 Experiment protocol

Each trial was done by a different subject and subjects were asked not to exercise or otherwise exert their arm for a full 24 hours prior to testing. During experimental trials, each subject was allowed to rest in between data measurements and disconnected from the data gathering devices, though the Sun electrodes remained on the arms of each subject for the duration of the testing period.

### 4.3 Results

**Figure 4:**
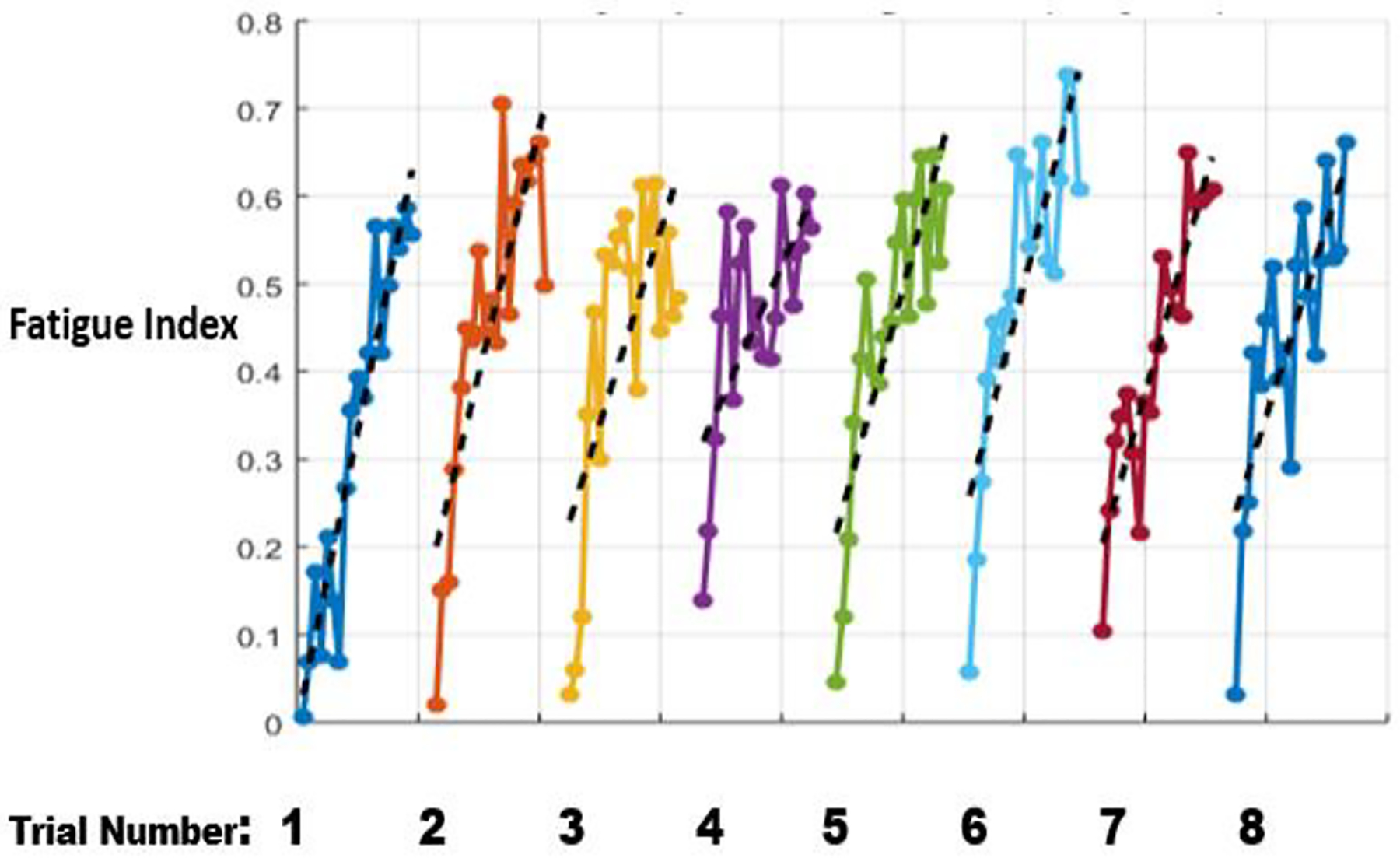
Fatigue Index for Same-day Repetitive Trial (subject 1). Each solid dot represents 1 FI point, and back dash line is the linear fit of the corresponding set of FI points

Figure 4 shows evolution of FIs of the relevant subject observed during the SRT. Inspection of fatigue patterns at the beginning of each repetition shows that each time, subject’s FI starts at about zero, indicating the that the current NMS model returned very close to the fresh model, which was trained using only the initial 20 seconds of the first trial. In addition, it is visible that during each exercise section, FIs show increasing trends, indicating increasing departure of NMS system performance away from the fresh model. More formally, we observed a statistically significant increasing linear trend in FIs corresponding to each session (utilizing tests proposed by Meals *et al*., 2011). Given the character and time-scale of the experiment, these changes can clearly be seen as NMS performance degradation caused by the fatiguing process.

**Figure 5:**
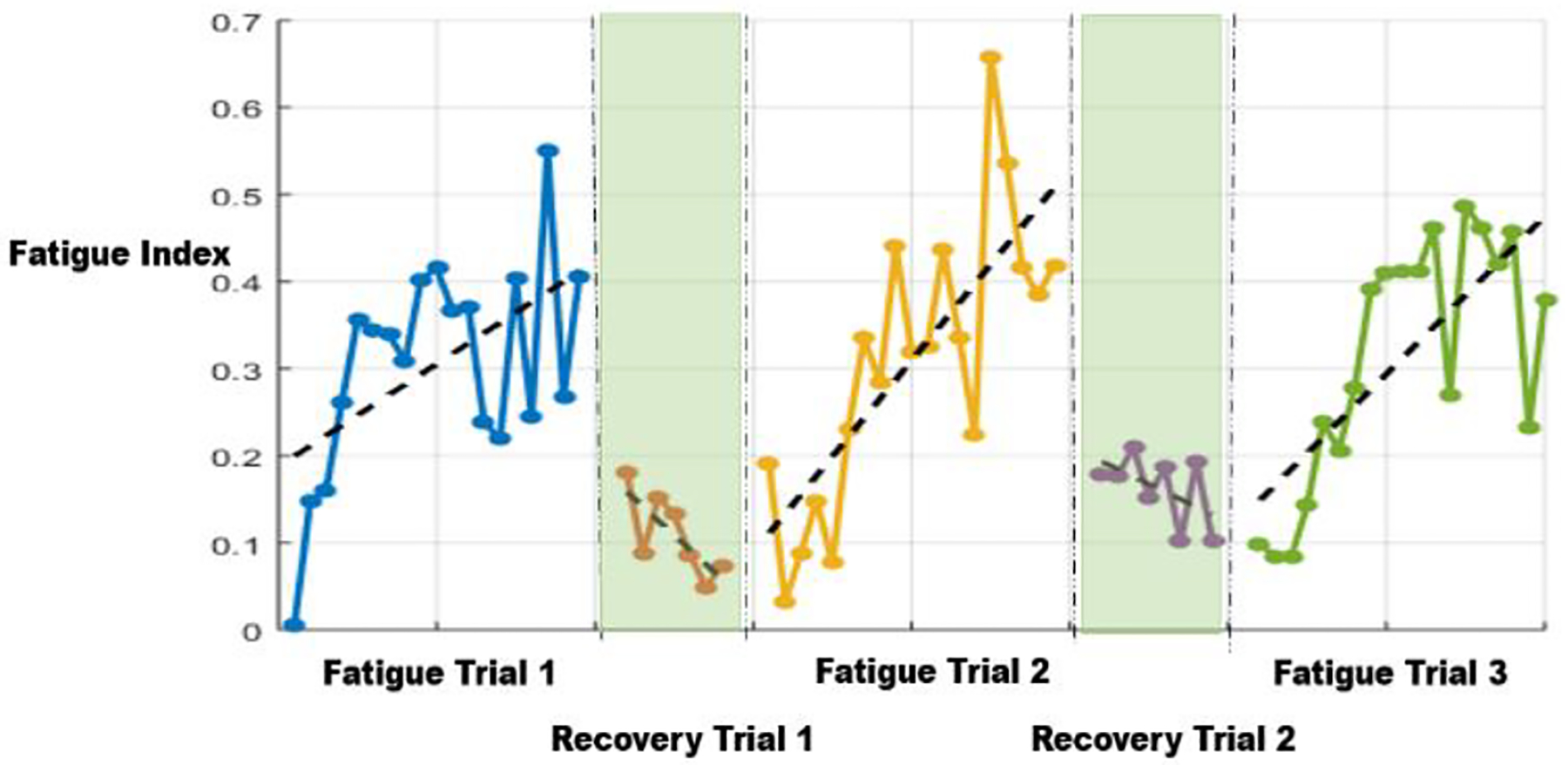
Fatigue Index for Repetitive Fatigue and Recovery Trail (subject 3).

Evolution of the FIs observed as the relevant subject performed RFRT is plotted in Figure 5. Once again, a clear increasing trend could be observed in the FIs during fatiguing portions of the trial. Furthermore, during the recovery portions of RFRT, one can observe decreasing trends in the FIs, illustrating recoveries of NMS system performance toward the fresh and unfatigued state. More formally, statistically significant linear increasing trends were observed during each fatiguing portion of the trial, and statistically significant linear decreasing trends could be seen during recovery portions of the trial. Such patterns of FIs increasing during fatigue trial and decreasing during recovery trials were observed for all subjects.

**Figure 6:**
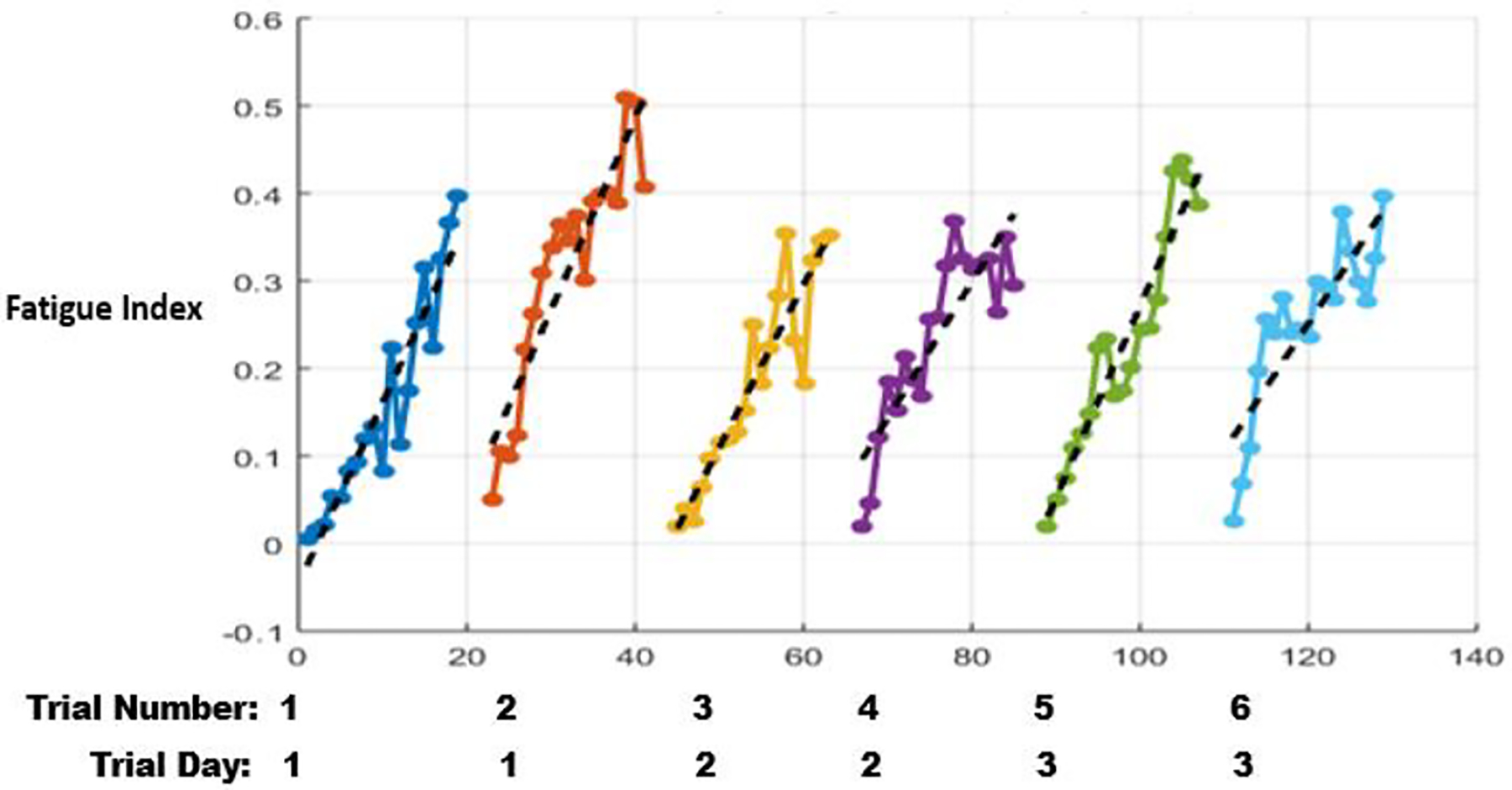
Fatigue Index for the Multi-day Repetitive Trial.

Finally, evolution of FIs observed as the relevant subject performed the MRT is shown in Figure 6. Since the subject is able to carry tattoo-like sensor for days, the model-based method is able to reliably capture the increasing FI patterns during the fatiguing portions of the trial, as well as recovered NMS states at the beginning of each session. From inspection of FI evolution in Figure 6, it is evident that subject’s fatigue pattern is consistent throughout the 3 consecutive days even though the corresponding fresh model is trained using only the initial dataset on the first day of the experiment. of the experiment.

## 5. Conclusions and Future Work

This paper advances the capabilities of stretchable electronics in the field of long-term repeatable and reliable monitoring of performance of a human NMS system. We describe a unique method of manufacturing stretchable skin-compliant sensors for long-term, noninvasive collection of human body physiological signals, as well as a new application for those sensors for model-based tracking of performance of a human NMS system.

The new devices for collection of EMG signals produced using the Cut-and-Paste manufacturing method are skin-compliant and can remain attached to the subject over multiple days, which enables a more consistent and reliable long-term data collection, compared to standard electrodes. The model-based performance monitoring method is based on building and tracking a dynamic model that relates EMG signatures and relevant NMS outputs (angles, angular velocities, forces). A simple Autoregressive Moving Average Model with Exogenous Inputs (ARMAX) form of the NMS model is employed, with instantaneous EMG intensities, frequencies, second order moments and entropies used as exogenous model inputs. A Fatigue Index (FI) indicating a Kulbeck-Leibler distance between the distribution of modeling errors observed at any given time and that of errors observed when the subject is fresh was used to quantitatively evaluate the relevant NMS performance at that time.

Three different experiments were conducted, involving hand gripping activities with subjects fatiguing and resting multiple times, including a trial that lasted multiple days, with new electrode remaining attached to the subject throughout that time. The relevant NMS system performance was evaluated using data from the forearm flexor EMG and the corresponding hand grip force. Throughout all trials, subjects showed consistent patterns of increasing FIs during exercising portions of the trials, as well as decreasing FIs during recovery portions. Furthermore, even though a very limited amount of data was used to training of the fresh models, it was noticed that after each resting session, the FIs consistently returned close to zero, showing a remarkably reliable and repeatable recovery of the relevant NMS system (no engineering machine recovers as reliably after maintenance, as the human NMS system studied here appears to recover after rest).

As for directions for future research, two major aspects of this study can be furtherly improved. Firstly, even if subjects are able to wear tattoo-like sensor during rest section without wiring constraint, the wire connection is still required during an exercise session. Advancement towards tattoo-like sensors with wireless data transmission capabilities is desirable since it enables free motion exercise and real-time monitoring. Also, integrating this unique sensor to current data collection platform can also be beneficial for further study of human body monitoring. Another aspect is the improvement of NMS system modeling. In this study, an essentially linear and very tractable ARMAX model is used to track the relevant NMS performance. This was possible because the system and function that were studied are rather simple. For more complex NMS systems with more elaborate and free joints and limbs movements, a non-linear model of the relevant NMS dynamics would be more appropriate.

In the long run, non-intrusive sensors and system-based approaches, such as those presented in this paper, should facilitate continuous long-term monitoring of performance of larger, more consequential portions of human NMS system. Such capabilities could improve health care by facilitating systematic and customized therapies for NMS injuries during which progress and effectiveness of any therapy could be tracked and perhaps optimized. In addition, in athletics, capabilities of quantitative long-term monitoring of NMS performance would lead to customized training and resting schedules, tailored to avoid injuries and maximize performance a specific athlete.

## Footnotes

1 Please note that the vector $\vec{X}(t)$ of EMG signatures is delayed by one time-sample to account for the fact that muscle activity does not instantaneously produce forces (to preserve causality of the resulting dynamic system).

## List of Abbreviations

AIC: Akaike Information Criterion
ARMAX: Autoregressive Moving Average
model: Model with exogenous inputs model
EMG: Electromyography
FI: Fatigue Index
GEM: Goal Equivalent Manifold
HGF: Hand grip force
MRT: Multi-day Repetitive Trial
MVC: Maximum Voluntary Contraction
NMS: Neuromusculoskeletal System
RFRT: Repetitive Fatigue and Recovery Trial
sEMG: Surface electromyography
SRT: Same-day Repetitive Trial
TFD: Time-frequency distribution

## References

[R1] Akaike H (1974). A new look at the statistical model identification. IEEE Transactions on Automatic Control, 19(6), 716–723

[R2] Allison GT, & Fujiwara T (2002). The relationship between EMG median frequency and low frequency band amplitude changes at different levels of muscle capacity. Clinical Biomechanics, 17(6), 464–469. 12135548 10.1016/s0268-0033(02)00033-5

[R3] Bilodeau M, Schindler-Ivens S, Williams DM, Chandran R, & Sharma SS (2003). EMG frequency content changes with increasing force and during fatigue in the quadriceps femoris muscle of men and women. Journal of Electromyography and Kinesiology, 13(1), 83–92. 12488090 10.1016/s1050-6411(02)00050-0

[R4] Cohen L (1995). Time-frequency analysis (Vol. 778). Prentice hall.

[R5] Dimitrov GV, Arabadzhiev TI, Mileva KN, Bowtell JL, Crichton N, & Dimitrova NA (2006). Muscle fatigue during dynamic contractions assessed by new spectral indices. Medicine and science in sports and exercise, 38(11), 1971. 17095932 10.1249/01.mss.0000233794.31659.6d

[R6] Dingwell JB, Napolitano DF, & Chelidze D (2007). A nonlinear approach to tracking slow-time-scale changes in movement kinematics. Journal of biomechanics, 40(7), 1629–1634. 16920121 10.1016/j.jbiomech.2006.06.019

[R7] Disselhorst-Klug C, Schmitz-Rode T, & Rau G (2009). Surface electromyography and muscle force: limits in sEMG–force relationship and new approaches for applications. Clinical biomechanics, 24(3), 225–235. 18849097 10.1016/j.clinbiomech.2008.08.003

[R8] Gates DH, & Dingwell JB (2008). The effects of neuromuscular fatigue on task performance during repetitive goal-directed movements. Experimental Brain Research, 187(4), 573–585. 18327575 10.1007/s00221-008-1326-8 PMC2825378

[R9] Gerdle B, Larsson B, & Karlsson S (2000). Criterion validation of surface EMG variables as fatigue indicators using peak torque: a study of repetitive maximum isokinetic knee extensions. Journal of Electromyography and Kinesiology, 10(4), 225–232. 10969195 10.1016/s1050-6411(00)00011-0

[R10] Jeong J, & Williams WJ (1992). Kernel design for reduced interference distributions. IEEE Transactions on Signal Processing, 40(2), 402–412.

[R11] Kabiri Ameri S, Ho R, Jang H, Tao L, Wang Y, Wang L, … & Lu N (2017). Graphene electronic tattoo sensors. ACS nano, 11(8), 7634–7641. 28719739 10.1021/acsnano.7b02182

[R12] Keynes RD, Aidley DJ, & Huang CLH (2001). Nerve and muscle (p. 126). New York:: Cambridge University Press.

[R13] Kuiken TA, Lowery MM, & Stoykov NS (2003). The effect of subcutaneous fat on myoelectric signal amplitude and cross-talk. Prosthetics and orthotics international, 27(1), 48–54. 12812327 10.3109/03093640309167976

[R14] Lu N, Wang X, Suo Z, & Vlassak J (2007). Metal films on polymer substrates stretched beyond 50%. Applied Physics Letters, 91(22), 221909.

[R15] Madden Kaci, “Monitoring Human Neuromusculoskeletal System Performance during Spacesuit Glove Use: A Pilot Study,” submitted to IEEE Aerospace Conference, Sky, Montana, March 3–10, 2017.

[R16] Matusita K (1955). Decision rules, based on the distance, for problems of fit, two samples, and estimation. The Annals of Mathematical Statistics, 631–640.

[R17] Meals DW, Spooner J, Dressing SA, & Harcum JB (2011). Statistical analysis for monotonic trends, Tech Notes 6, November 2011. Developed for US Environmental Protection Agency by Tetra Tech, Inc., Fairfax, VA.

[R18] Musselman M, & Djurdjanovic D (2012). Time–frequency distributions in the classification of epilepsy from EEG signals. Expert Systems with Applications, 39(13), 11413–11422.

[R19] Musselman M, Gates D, & Djurdjanovic D (2017, March). System based monitoring of a neuromusculoskeletal system using divide and conquer type models. In Aerospace Conference, 2017 IEEE (pp. 1–12). IEEE.

[R20] Pandit SM, & Wu SM (1983). Time series and system analysis with applications (Vol. 272). New York: Wiley.

[R21] Potvin JR (1997). Effects of muscle kinematics on surface EMG amplitude and frequency during fatiguing dynamic contractions. Journal of Applied Physiology, 82(1), 144–151. 9029209 10.1152/jappl.1997.82.1.144

[R22] Vøllestad NK (1997). Measurement of human muscle fatigue. Journal of neuroscience methods, 74(2), 219–227. 9219890 10.1016/s0165-0270(97)02251-6

[R23] Wang Y, Qiu Y, Ameri SK, Jang H, Dai Z, Huang Y, & Lu N (2018). Low-cost, μm-thick, tape-free electronic tattoo sensors with minimized motion and sweat artifacts. npj Flexible Electronics, 2(1), 6.

[R24] Yamagami M, Peters KM, Milovanovic I, Kuang I, Yang Z, Lu N, & Steele KM (2018). Assessment of Dry Epidermal Electrodes for Long-Term Electromyography Measurements. Sensors, 18(4), 1269 10.3390/s18041269 PMC5948629 29677129

[R25] Yang S, Chen YC, Nicolini L, Pasupathy P, Sacks J, Su B, … & Schnyer D (2015). “Cut-and-Paste” Manufacture of Multiparametric Epidermal Sensor Systems. Advanced Materials, 27(41), 6423–6430 26398335 10.1002/adma.201502386

